# Determinants of High-School Dropout: A Longitudinal Study in a Deprived Area of Japan

**DOI:** 10.2188/jea.JE20170163

**Published:** 2018-11-05

**Authors:** Takahiro Tabuchi, Sho Fujihara, Tomohiro Shinozaki, Hiroyuki Fukuhara

**Affiliations:** 1 Cancer Control Center, Osaka International Cancer Institute, Osaka, Japan; 2 Urban Research Plaza, Osaka City University, Osaka, Japan; 3 Center for Social Research and Data Archives, Institute of Social Science, The University of Tokyo, Tokyo, Japan; 4 Department of Biostatistics, School of Public Health, The University of Tokyo, Tokyo, Japan; 5 Department of Economics, Osaka City University, Osaka, Japan

**Keywords:** determinants of high-school dropout, tardy arrival, daily smoking, Japan

## Abstract

**Background:**

Our objective in this study was to find determinants of high-school dropout in a deprived area of Japan using longitudinal data, including socio-demographic and junior high school-period information.

**Methods:**

We followed 695 students who graduated the junior high school located in a deprived area of Japan between 2002 and 2010 for 3 years after graduation (614 students: follow-up rate, 88.3%). Multivariable log-binomial regression models were used to calculate the prevalence ratios (PRs) for high-school dropout, using multiple imputation (MI) to account for non-response at follow-up.

**Results:**

The MI model estimated that 18.7% of students dropped out of high school in approximately 3 years. In the covariates-adjusted model, three factors were significantly associated with high-school dropout: ≥10 days of tardy arrival in junior high school (PR 6.44; 95% confidence interval [CI], 1.69–24.6 for “10–29 days of tardy arrival” and PR 8.01; 95% CI, 2.05–31.3 for “≥30 days of tardy arrival” compared with “0 day of tardy arrival”), daily smoking (PR 2.01; 95% CI, 1.41–2.86) and severe problems, such as abuse and neglect (PR 1.66; 95% CI, 1.16–2.39). Among students with ≥30 days of tardy arrival in addition to daily smoking or experience of severe problems, ≥50% high-school dropout rates were observed.

**Conclusions:**

Three determinants of high-school dropout were found: smoking, tardy arrival, and experience of severe problems. These factors were correlated and should be treated as warning signs of complex behavioral and academic problems. Parents, educators, and policy makers should work together to implement effective strategies to prevent school dropout.

## INTRODUCTION

Educational attainments, usually achieved in youth, are strongly associated with later employment, type of occupation, and income in adulthood and also affect health status.^1^^,^^2^ Therefore, education is a representative social determinant of health worldwide and in Japan.^3^^,^^4^ Secondary education in the Japanese system comprises 3 years of compulsory junior high school and 3 years of non-compulsory high school. The first grade of junior high school corresponds to seventh grade in the United States, and the third grade of high school corresponds to the 12th grade. In recent decades, more than 95% of junior-high-school students continued to high school in Japan^5^; however, approximately 2 percent of high school students drop out of school within a year.^6^ More than 50% of high-school dropouts started their first job as non-standard employees, while this percentage was approximately 20% for high school graduates who had not attended college.^7^

Adolescence is an important period for human development, but it is also often a rebellious period.^8^ Adolescents’ behaviors can deviate from social or legal norms, and these behaviors —such as tobacco, alcohol, or drug use, truancy, and unsafe sex— tend to co-occur, resulting in poor academic outcomes, such as school dropout.^9^^,^^10^ These risky behaviors are also correlated with their environment, such as the socioeconomic status of their households or place of residence (ie, deprived or affluent area).^1^ Previous studies of children and adolescents in the United States found that growing up in deprived neighborhoods is associated with many social problems, such as delinquency, low intelligence quotient, and dropping out.^11^^,^^12^ A previous study in Japan reported that more than 30% of students with the lowest education level (lowest quintile of entrance examination average score) dropped out of high school, while less than 3% with the highest education level (highest quintile) dropped out.^13^ However, this study did not account for other factors, including information from junior high school. Empirical longitudinal studies that examined the risk factors for high-school dropout have mostly been conducted in the United States or Europe and are scarce in Japan,^9^^,^^14^^–^^16^ especially in deprived areas. Our objective in this study was to find determinants of high-school dropout in a deprived area in Japan using longitudinal data, including socio-demographic and junior high school-period information. This will provide insights into preventive measures for policy-makers and other professionals.

## METHODS

### Data

The target population of the study were all students in a junior high school located in a northern part of Nishinari ward, Osaka, Japan.^17^ According to the 2005 census, the ward is one of the most deprived areas, with a higher unemployment rate (22.4%) than the overall Japanese total (approximately 5%). Providing continuous support for all students, including vulnerable students, was considered to be very important, not only during compulsory attendance at junior high school but also after graduation. To take care of the students and to conduct this study, we utilized our personal and organized relationship within the junior high school and its neighboring area organizations, including junior high school, local volunteer groups, local NGOs, and local government. Information on students’ socio-demographic and school life-related factors were collected between 2000 and 2013 from various school records, repeated questionnaires, or personal communication (eg, with students, teachers, parents, and guardians). For example, total days of tardy arrival were calculated from the attendance record of the junior high school. Daily smoking was determined using the lifestyle guidance record by the teacher and personal communications between teachers and students. These data were integrated as explanatory variables: sex (boy or girl), calendar period of junior high school graduation (2002–2004, 2005–2007, or 2008–2010), household economic status (receiving public assistance for life [seikatsu-hogo in Japanese], public assistance for school life [shugaku-enjo in Japanese], or other), family structure (living with mother and father or living with either or neither parent), nationality (Japanese or other), total days of tardy arrival in junior high school during the 3-year period (quartiles of 0 day, 1–9 days, 10–29 days, or ≥30 days), total days of absence in junior high school during the 3-year period (quartiles of 0 day, 1–2 days, 3–9 days, or ≥10 days), smoking status at 9th grade (daily smoker or non-daily smoker), experience of severe problems (such as abuse and neglect), and achieved academic level (level of entrance examination difficulty of the high school the student attended was used as a proxy variable for the student’s achieved academic level). Experience of severe problems was collected according to student’s records from areal case-conference by municipality or NGOs. Severe problems included child abuse, neglect, bullying, poverty, and risky behaviors, such as arson, violence, and robbery. Most cases had many problems concurrently, such as economic problems, aggravated interpersonal relations, mental and physical illness, and trouble in school, family, and life. Once a student was recognized as a case (for case-conference) if his/her comprehensive problems were complex, classifying his/her problem to one reason was not appropriate. However, the most critical reason (based on the conference consensus) was recorded for the dataset. Thus, experience of severe problems was not classified by each specific reason but just by the most important one and then dichotomized (0 or 1).

The standard definition of public assistance in Japan is available elsewhere.^18^^,^^19^ Tardy arrival was defined as arrival at the school gate after 8:30 am. Achieved academic level was defined using the Japanese high school “deviation value” system.^20^ Japanese high schools are highly stratified, and the within-stratum rank of students was highly correlated with their academic performance.^21^ A trial exam is given as a guide to make it easier for students to decide on which schools’ test to take when matriculating on to high school; this shows “deviation value”. Four categories for the value (lowest [≤39], 2nd [40–49], 3rd [50–59], and highest [≥60]) were used. The information was collected for students who graduated junior high school between 2002 and 2010 and was used as baseline data for the longitudinal assessment.

**Figure 1.**
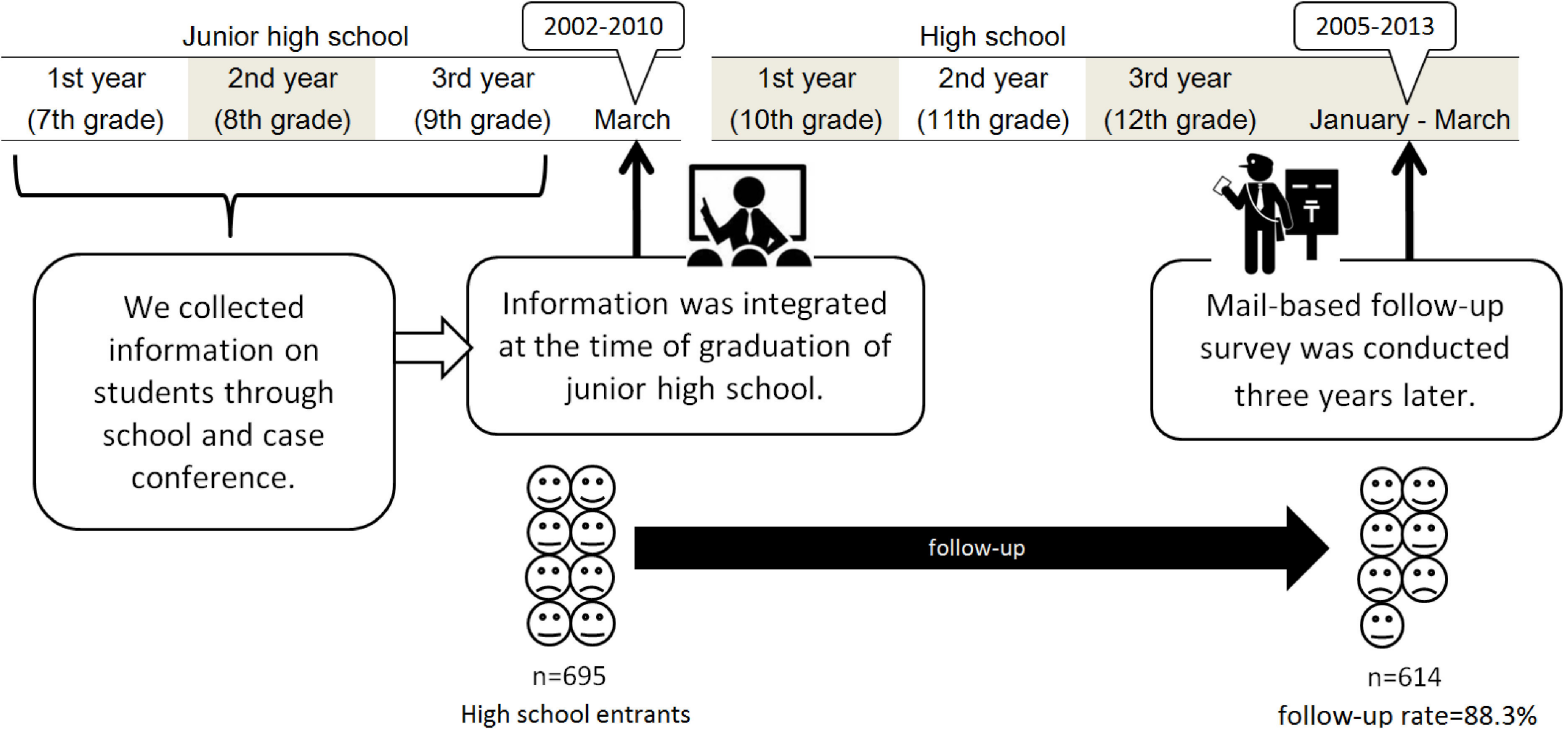
Study design with time course

Study design with the time course is shown in Figure 1. Students were followed up by mail-based surveys to collect information on high-school dropout approximately 3 years after junior high school graduation (ie, from January to March, corresponding with the last three months of 12th grade). Participants were asked whether they dropped out of high school. According to the answer to this question, high-school dropout was dichotomized as an outcome variable. Registered absence from school and studying abroad were not defined as dropout. Students who dropped out and moved to another high school later were defined as dropout according to a previous study.^22^ To increase the response rate for the follow-up survey, questionnaires for the survey were sent with a letter that students had written to their 3-year older selves (these letters may capture the attention of students). These letters were prepared immediately before junior high school graduation. At every chance during junior high school, students were informed that participation was voluntary and their answers were confidential. When students graduated junior high school, their consent to the follow-up survey was obtained. As no student declined to participate in the study, data from all students who graduated junior high school was analyzed in the study (baseline response rate = 100%). Analysis of the research was approved by the institutional review boards of Osaka City University (2012).

### Statistical analyses

Of students (*n* = 731) who consented to participate in the study and graduated junior high school between 2002 and 2010, 95.1% (*n* = 695) attended high school, 3.4% (*n* = 25) were working (regular employment), and 1.5% (*n* = 11) were undecided (including part-time employment or students who had failed an entrance examination for high school). The eligible 695 high school students were followed up for evaluation of their high-school dropout (responses were available from 614 students; follow-up rate = 88.3%).

Spearman correlation coefficients were calculated to assess the association between characteristics (explanatory variables). Log-binomial regression models were used to calculate the prevalence ratios (PRs) and 95% confidence intervals (CIs) for high-school dropout, as the outcome occurred in more than 10% of the sample.^23^^,^^24^ In some instances, the models did not converge, so we used log-Poisson models, which provide consistent but not fully efficient estimates of the PRs (ie, the confidence intervals are slightly wider).^24^^,^^25^ Univariable and multivariable analyses were used to document the crude and adjusted relationship between independent explanatory variables and high-school dropout. Characteristics of subjects who participated in the study may differ from those who did not respond at follow-up survey (eTable 1). To account for non-response at the follow-up survey, we used two imputation methods in addition to a non-imputed analysis (complete case analysis). First, we used multivariable multiple imputation (MI) of missing data.^26^^,^^27^ For the imputation model, we included all subject characteristics as well as the outcome. We generated 100 imputed datasets and combined estimates across these using Rubin’s rules. Details of the imputation model are available in the supplemental information (eTable 2). Second, we used a monotone imputation of missing data for the outcome (high-school dropout), while missing explanatory variables were not imputed. In the monotone model, we assumed that missing follow-up information indicated high-school dropout (coding missing = dropout). This is because cases missing at follow-up could be a group at high risk of dropout, although they refused to participate for a variety of reasons. Cases with missing value (outcome or explanatory variable) were excluded from the regression analyses. Results using MI are shown as main results.

Probability values for statistical tests were two-tailed, and *P* < 0.05 was considered statistically significant. All analyses were performed using SAS version 9.3 (SAS Institute, Cary, NC, USA).

## RESULTS

Characteristics of the study subjects and the high-school dropout rates are shown in Table 1. During the 3-year follow-up, 18.7% of students had dropped out of high school in the MI model (15.0% in complete-case model and 26.6% in a monotone imputation model). Some characteristics show high dropout rates of more than 20% in the MI model: boy (20.3%), public assistance for life (24.1%), not living with two parents (20.6%), having ≥10 days of tardy arrival in junior high school (22.8% for 10–29 days and 42.9% for ≥30 days), having ≥3 days of absence in junior high school (23.6% for 3–9 days and 34.0% for ≥10 days), daily smoking (48.2%), experience of a severe problems (39.4%), and lowest high school level (27.4%).

**Table 1.** Basic characteristics and high-school dropout in study subjects

Characteristics	*n* (%)	High-school dropout			

		Pre-imputation		Post-multiple imputation (imputed 100 datasets)	Post-monotone imputation^a^

		*n* (%)	Missing, *n* (%)	%	%
Total	695 (100.0)	104 (15.0)	81 (11.7)	18.7	26.6

***Socio-demographic factors***					
Sex					
Boy	387 (55.7)	61 (15.8)	58 (15.0)	20.3	30.7
Girl	308 (44.3)	43 (14.0)	23 (7.5)	16.6	21.4
Year of high school admission					
2002–2004	216 (31.1)	25 (11.6)	41 (19.0)	19.9	30.6
2005–2007	231 (33.2)	36 (15.6)	30 (13.0)	17.8	28.6
2008–2010	248 (35.7)	43 (17.3)	10 (4.0)	18.4	21.4
Economic status of the household					
Public assistance for life (“Seikatsu-hogo”)	137 (19.7)	26 (19.0)	16 (11.7)	24.1	30.7
Public assistance for school (“Shugaku-enjo”)	340 (48.9)	51 (15.0)	48 (14.1)	19.2	29.1
Others	218 (31.4)	27 (12.4)	17 (7.8)	14.5	20.2
Family structure					
Living with two parents	414 (59.6)	58 (14.0)	50 (12.1)	17.4	26.1
Living with either or neither parent	281 (40.4)	46 (16.4)	31 (11.0)	20.6	27.4
Nationality					
Japan	645 (92.8)	101 (15.7)	76 (11.8)	19.5	27.4
Others	50 (7.2)	3 (6.0)	5 (10.0)	8.3	16.0

***Junior high school-life-related factors***					
Total days of tardy arrival in junior high school					
0 day	126 (18.1)	2 (1.6)	10 (7.9)	2.3	9.5
1–9 days	243 (35.0)	8 (3.3)	23 (9.5)	5.7	12.8
10–29 days	133 (19.1)	24 (18.0)	19 (14.3)	22.8	32.3
≥30 days	192 (27.6)	70 (36.5)	28 (14.6)	42.9	51.0
Missing	1 (0.1)	0 (0.0)	1 (100.0)		
Total days of absence in junior high school					
0 day	158 (22.7)	7 (4.4)	14 (8.9)	6.5	13.3
1–2 days	174 (25.0)	11 (6.3)	15 (8.6)	8.7	14.9
3–9 days	180 (25.9)	37 (20.6)	22 (12.2)	23.6	32.8
≥10 days	176 (25.3)	49 (27.8)	29 (16.5)	34.0	44.3
Missing	7 (1.0)	0 (0.0)	1 (14.3)		
Daily smoking					
No	543 (78.1)	43 (7.9)	57 (10.5)	10.4	18.4
Yes	152 (21.9)	61 (40.1)	24 (15.8)	48.2	55.9
Severe problems such as abuse and neglect					
No	613 (88.2)	76 (12.4)	72 (11.7)	15.9	24.1
Yes	82 (11.8)	28 (34.1)	9 (11.0)	39.4	45.1
Achieved academic level					
Lowest	376 (54.1)	84 (22.3)	48 (12.8)	27.4	35.1
2nd	165 (23.7)	12 (7.3)	16 (9.7)	9.7	17.0
3rd	99 (14.2)	7 (7.1)	13 (13.1)	9.3	20.2
Highest	55 (7.9)	1 (1.8)	4 (7.3)	2.9	9.1

^a^Coding missing = droptout.

The correlation matrix among explanatory variables used in this study is shown in Table 2. Most factors, such as household economic status, family structure, total days of tardy arrival, total days of absence, and daily smoking, showed significant correlations with each other (≥5 combinations of significant correlations were observed in these factors), while correlation was relatively weak for sex, calendar period, and nationality.

**Table 2.** Correlation matrix of explanatory variables

	Explanatory variables	Spearman correlation coefficients									

		*i*	*ii*	*iii*	*iv*	*v*	*vi*	*vii*	*viii*	*ix*	*x*
*i)*	Sex	1	0.03	0.01	0.04	−0.05	−0.07	0.05	**−0.08**	**0.08**	−0.03
*ii)*	Year of high school admission		1	−0.07	−0.01	−0.06	0.06	0.02	−0.05	**0.16**	−0.01
*iii)*	Economic status of the household			1	**0.39**	−0.05	**0.10**	**0.12**	**0.09**	**0.27**	**0.23**
*iv)*	Family structure				1	**−0.14**	**0.12**	**0.16**	**0.10**	**0.17**	**0.22**
*v)*	Nationality					1	−0.07	**−0.14**	**−0.12**	0.02	**−0.12**
*vi)*	Total days of tardy arrival						1	**0.50**	**0.45**	**0.15**	**0.38**
*vii)*	Total days of absence							1	**0.32**	**0.26**	**0.31**
*viii)*	Daily smoking								1	**0.16**	**0.27**
*ix)*	Severe problems such as abuse and neglect									1	**0.19**
*x)*	Achieved academic level										1

**Bold** indicates statistical significance of <0.05.

Results of the univariable and multivariable analyses for high-school dropout are shown in Table 3. In both the univariable and multivariable models in all three settings (pre-imputation, post-MI, and post monotone imputation), three baseline factors were significantly associated with later high-school dropout: having ≥10 days of tardy arrival in junior high school (PR 6.44; 95% CI, 1.69–24.6 for “10–29 days of tardy arrival” and PR 8.01; 95% CI, 2.05–31.3 for “≥30 days of tardy arrival” compared with “0 day of tardy arrival”), daily smoking (PR 2.01; 95% CI, 1.41–2.86) and experience of severe problems (PR 1.66; 95% CI, 1.16–2.39) in the MI models. Regarding other variables, child’s household economic status, days of absence in junior high school, and achieved academic levels showed significant association with high-school dropout in most univariable models. However, after multivariable adjustments, these factors were no longer significantly associated.

**Table 3.** Unadjusted and adjusted relative risk for high-school dropout, pre-imputed and post-imputed results

Characteristics	Pre-imputation (complete case)		Post-multiple imputation (imputed 100 datasets)		Post-monotone imputation^a^	
		
	Unadjusted RR (95% CI)	Adjusted RR (95% CI)^b^	Unadjusted RR (95% CI)	Adjusted RR (95% CI)^b^	Unadjusted RR (95% CI)	Adjusted RR (95% CI)^b^
***Socio-demographic factors***						
Sex						
Boy	1 (reference)	1 (reference)	1 (reference)	1 (reference)	1 (reference)	1 (reference)
Girl	0.81 (0.57–1.16)	0.82 (0.58–1.15)	0.82 (0.59–1.14)	0.86 (0.63–1.17)	**0.70 (0.54–0.90)**	**0.73 (0.57–0.93)**
Year of high school admission						
2002–2004	1 (reference)	1 (reference)	1 (reference)	1 (reference)	1 (reference)	1 (reference)
2005–2007	1.25 (0.78–2.00)	0.90 (0.59–1.36)	0.94 (0.42–2.11)	0.81 (0.37–1.75)	0.94 (0.70–1.25)	0.85 (0.64–1.12)
2008–2010	1.26 (0.80–1.99)	0.98 (0.65–1.48)	0.98 (0.45–2.13)	0.85 (0.39–1.85)	**0.70 (0.51–0.96)**	**0.66 (0.49–0.89)**
Economic status of the household						
Public assistance for life (“Seikatsu-hogo”)	1.60 (0.98–2.61)	1.04 (0.64–1.70)	**1.67 (1.03–2.70)**	1.13 (0.69–1.87)	**1.52 (1.05–2.19)**	1.19 (0.83–1.73)
Public assistance for school (“Shugaku-enjo”)	1.30 (0.85–2.00)	1.09 (0.75–1.57)	1.33 (0.88–1.99)	1.07 (0.75–1.54)	**1.44 (1.06–1.97)**	1.24 (0.94–1.64)
Others	1 (reference)	1 (reference)	1 (reference)	1 (reference)	1 (reference)	1 (reference)
Family structures						
Living with two parents	1 (reference)	1 (reference)	1 (reference)	1 (reference)	1 (reference)	1 (reference)
Living with either or neither parent	1.15 (0.81–1.64)	0.84 (0.60–1.17)	1.18 (0.85–1.64)	0.80 (0.56–1.13)	1.05 (0.82–1.35)	0.78 (0.61–1.00)
Nationality						
Japan	1 (reference)	1 (reference)	1 (reference)	1 (reference)	1 (reference)	1 (reference)
Others	0.38 (0.12–1.14)	0.62 (0.21–1.82)	0.41 (0.15–1.15)	0.66 (0.25–1.73)	0.58 (0.31–1.11)	0.75 (0.38–1.47)

***Junior high school-life-related factors***						
Total days of tardy arrival in junior high school						
0 day	1 (reference)	1 (reference)	1 (reference)	1 (reference)	1 (reference)	1 (reference)
1–9 days	2.11 (0.46–9.77)	1.67 (0.35–7.96)	2.53 (0.64–9.93)	2.05 (0.51–8.25)	1.34 (0.71–2.52)	1.13 (0.59–2.16)
10–29 days	**12.21 (2.95–50.47)**	**7.31 (1.66–32.20)**	**10.32 (2.80–38.08)**	**6.44 (1.69–24.64)**	**3.39 (1.88–6.13)**	**2.36 (1.25–4.43)**
≥30 days	**24.76 (6.20–98.93)**	**8.93 (1.95–40.99)**	**19.47 (5.43–69.87)**	**8.01 (2.05–31.31)**	**5.36 (3.07–9.34)**	**2.93 (1.55–5.54)**
Total days of absence in junior high school						
0 day	1 (reference)	1 (reference)	1 (reference)	1 (reference)	1 (reference)	1 (reference)
1–2 days	1.42 (0.57–3.57)	1.06 (0.45–2.46)	1.37 (0.59–3.17)	1.02 (0.46–2.28)	1.12 (0.66–1.92)	0.90 (0.54–1.52)
3–9 days	**4.82 (2.22–10.46)**	2.00 (0.96–4.17)	**3.77 (1.70–8.35)**	1.70 (0.79–3.67)	**2.47 (1.57–3.87)**	1.49 (0.94–2.37)
≥10 days	**6.86 (3.21–14.63)**	1.77 (0.84–3.76)	**5.42 (2.60–11.28)**	1.47 (0.72–3.01)	**3.33 (2.17–5.13)**	1.43 (0.90–2.27)
Daily smoking						
No	1 (reference)	1 (reference)	1 (reference)	1 (reference)	1 (reference)	1 (reference)
Yes	**5.39 (3.84–7.55)**	**2.07 (1.46–2.94)**	**4.67 (3.30–6.60)**	**2.01 (1.41–2.86)**	**3.04 (2.42–3.81)**	**1.57 (1.23–2.00)**
Severe problems such as abuse and neglect						
No	1 (reference)	1 (reference)	1 (reference)	1 (reference)	1 (reference)	1 (reference)
Yes	**2.73 (1.91–3.90)**	**1.65 (1.15–2.37)**	**2.50 (1.64–3.81)**	**1.66 (1.16–2.39)**	**1.87 (1.42–2.47)**	**1.47 (1.11–1.95)**
Achieved academic level						
Lowest	1 (reference)	1 (reference)	1 (reference)	1 (reference)	1 (reference)	1 (reference)
2nd	**0.31 (0.18–0.56)**	0.71 (0.41–1.24)	**0.35 (0.20–0.61)**	0.66 (0.39–1.12)	**0.48 (0.34–0.70)**	0.83 (0.59–1.17)
3rd	**0.32 (0.15–0.66)**	1.01 (0.49–2.06)	**0.33 (0.16–0.68)**	0.90 (0.46–1.74)	**0.58 (0.38–0.87)**	1.11 (0.73–1.70)
Highest	**0.08 (0.01–0.54)**	0.23 (0.04–1.38)	**0.10 (0.02–0.57)**	0.31 (0.06–1.63)	**0.26 (0.11–0.60)**	0.62 (0.26–1.47)

CI, confidence interval; RR, relative risk.

^a^Coding missing = dropout.

^b^Adjusted for all listed variables.

**Figure 2.**
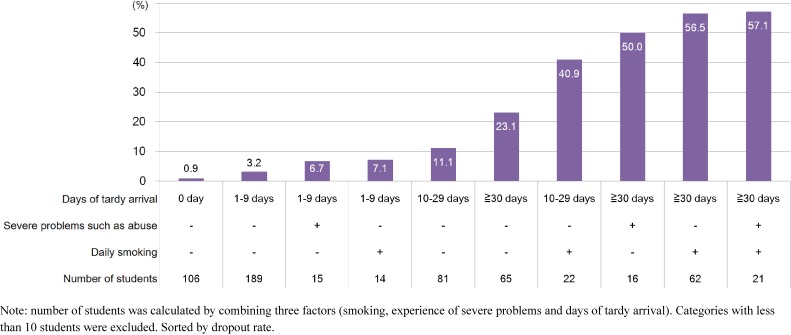
Dropout rate according to combination of smoking, experience of severe problems and days of tardy arrival. Pre-imputation dataset (complete case)

Focusing on the three variables which were significantly associated with high-school dropout in the adjusted models, dropout rate according to combinations of total days of tardy arrival, smoking status, and experience of severe problems are shown in Figure 2. Among those with ≥30 days of tardy arrival in addition to daily smoking or experience of severe problems, the high-school dropout rate was ≥50%. On the other hand, those with negative status for all three variables (having 0 days of tardy arrival, no daily smoking, and no experience of severe problems) showed a high-school dropout rate of less than 1% (0.9%). High-school dropout rates were less than 10% for those having 1–9 days of tardy arrival if students smoked daily or experienced severe problems (either one).

## DISCUSSION

The MI model estimated that 18.7% of students dropped out of high school during a period of approximately 3 years in a deprived area in Japan. During the study period (2000–2013), the high-school dropout rates within 1 year among all high-school students (corresponding 10–12th grade in the United States) ranged between 1.5% and 2.6% in Japan.^6^ Compared with these figures, a considerably higher high-school dropout rate was observed in the current study. However, compared with the figure from high school with the lowest education level (lowest quintile of entrance examination average score) (≥30%)^13^ as stated in the introduction, the dropout rate in the current study was lower, even in the monotone imputation model (26.6%).

Three factors were significantly associated with high-school dropout consistently in all multivariable models (MI, monotone-imputed and complete-case models): ≥10 days of tardy arrival in junior high school, daily smoking, and experience of severe problems. According to the numerical values of the PRs (see Table 3), ≥10 days of tardy arrival may have a greater effect on high-school dropout than other factors, including absence. This may be because absence comprises all absence, including unavoidable absence due to infectious diseases, which happen regardless of socioeconomic condition, whereas tardiness may often be derived from a disordered daily life. Students who are often late for school may not be able to keep up with lessons and drop out of high school. In this study, information on tardiness in high school was not obtained, but tardiness in junior high school may predict tardiness in high school.

As for smoking, the observed rate of daily smoking in junior-high-school students in this study (21.9% for both sexes; 24.8% for boys and 18.2% for girls) was much higher than that of a previous study among representative Japanese junior-high-school students,^28^ which found that 5.2% of male students smoked daily (1.8% for girls). The earlier people start smoking (eg, early teenage), the greater the harm. Although the smoking rates of junior-high-school students have been rapidly declining in Japan in the past 20 years,^29^ we need to prevent smoking in junior high school. In the USA 2013 Youth Risk Behavior Survey, the prevalence of current cigarette smoking was 10.2% among ninth graders.^30^ Previous epidemiologic research suggests that school dropout has been linked to mental health conditions, substance use, chronic health problems, and criminal behavior.^16^ However, longitudinal studies where smoking evidently preceded school dropout are scarce. In the current study, we found that daily smoking is a leading indicator for future risk of high-school dropout. When we find a student who smokes, we should not only support him or her to stop smoking but should also treat smoking as a sign of increased risk of future school dropout and, therefore, of the need to provide preventive care for his/her school life. This speculation may be supported by previous studies that found that smoking was associated with other indicators of academic problems, such as boredom or lack of connection to school.^31^^–^^33^

Regarding experience of severe problems, the experience included various types of social issues (child abuse, neglect, poverty, bullying, and truancy) and was significantly associated with high-school dropout. This is unsurprising, as these events often reflect various difficult aspects of the student’s socioeconomic condition. However, the adjusted PR of the experience was slightly lower than that of daily smoking (1.66 vs 2.01 in the MI model). This may indicate that daily smoking should be treated in the same way as experience of severe problems.

Adolescence is a period of human development marked by the co-occurrence of multiple risk taking behaviors, such as smoking. To date, in many previous studies, academic achievement level has been found to be associated with school dropout.^34^^,^^35^ In the current study, academic level was not significantly associated with dropout after covariates-adjustment, consistent with a previous study using tardy arrival as a covariate.^15^ Regardless of academic achievement levels, for example, students who smoke and have a rebellious attitude (a typical pattern of smoking)^36^ may defy school rules and teachers, so their school life may not be successful. Results highlight that these three determinants, which correlate with each other, may comprise a complex, maladaptive behavioral problem. Accounting for multiple risk factors and their interactions may be necessary to develop prevention programs.

### Limitations and strengths of the study

There are several limitations to this study. The study was conducted in a specific geographic area, a socially deprived environment in Japan, limiting the generalizability of the findings. The estimated dropout rate may be an overestimate, especially for the monotone imputation model. Some participants were lost to follow-up or withdrew consent; however, the statistical analysis was robust to missing data.^37^ Unmeasured factors may have biased the results. For example, information on the educational attainments of students’ parents was not available in the study. Finally, more research is needed to understand the socioeconomic, familial, and other school-related characteristics of adolescents. Despite these limitations, this study has the strengths of a prospective design, which allows examination of a detailed picture of high-school dropout, and the use of multiple covariate adjustments.

### Conclusion

High-school dropout will have a lifelong impact of lower income, difficulty finding employment, poorer health, and higher incarceration rates.^38^ Three factors were statistically significant determinants of high-school dropout: ≥10 days of tardy arrival in junior high school, daily smoking, and experience of severe problems. These factors significantly correlate with each other. They should be treated as warning signs of complex behavioral and academic problems. Parents, educators, and policy makers should work together to implement effective strategies to prevent school dropout.

## APPENDIX A. SUPPLEMENTARY DATA

eTable 1. Baseline characteristics of study subjects comparing responders with non-responders at follow-up survey

eTable 2. Variables included in the multiple imputation model
