# Supplementary material for: Determinants of High-School Dropout: A Longitudinal Study in a Deprived Area of Japan
Source: J Epidemiol. 2018 Nov 5;28(11):458–64. doi: 10.2188/jea.JE20170163 (PMC6192975; doi:10.2188/jea.JE20170163)
Supplement: Supplementary file 1 [file je-28-458-s001.pdf]

eTable 1. Baseline characteristics of study subjects comparing responders with non-responders at follow-up survey

	Responders n=614		Non-responders n=81		
Characteristics	n	%	n	%	P for difference*
Socio-demographic factors					
Sex					0.0027
Boy	329	53.6	58	71.6	
Girl	285	46.4	23	28.4	
Year of high school admission					<.0001
2002–2004	175	28.5	41	50.6	
2005–2007	201	32.7	30	37.0	
2008–2010	238	38.8	10	12.3	
Economic status of the household					0.0709
No assistance	201	32.7	17	21.0	
Public assistance for school ("Shugaku-enjo")	292	47.6	48	59.3	
Public assistance for life ("Seikatsu-hogo")	121	19.7	16	19.8	
Family structure					0.7188
Living with two parents	364	59.3	50	61.7	
Living with either or neither parent	250	40.7	31	38.3	
Nationality					0.7167
Japan	569	92.7	76	93.8	
Others	45	7.3	5	6.2	
Junior high school-life-related factors					
Total days of "tardy arrival" in junior high school					0.1471
0 day	116	18.9	10	12.3	
1–9 days	220	35.8	23	28.4	
10–29 days	114	18.6	19	23.5	
≥30 days	164	26.7	28	34.6	
Missing	0	0.0	1	1.2	
Total days of absence in junior high school					0.0887
0 day	144	23.5	14	17.3	
1–2 days	159	25.9	15	18.5	
3–9 days	158	25.7	22	27.2	
≥10 days	147	23.9	29	35.8	
Missing	6	1.0	1	1.2	
Daily smoking					0.0855
No	486	79.2	57	70.4	
Yes	128	20.8	24	29.6	
Experience of severe problems such as abuse					0.6389
No	541	88.1	72	88.9	
Yes	73	11.9	9	11.1	
Achieved academic level					0.5517
Lowest	328	53.4	48	59.3	
2nd	149	24.3	16	19.8	
3rd	86	14.0	13	16.0	
Highest	51	8.3	4	4.9	

* Fisher exact tests.

eTable 2. Variables included in the multiple imputation model

Variable	Type of variable	Model used to predict missing data in this variable	How variable was entered in model to predict missing data in other variables
Dropout (outcome variable in the study)	Binary	Logistic regression	Binary
Total days of absence	Continuous	Linear regression	Continuous
Total days of tardy arrival	Continuous	Linear regression	Continuous
Achieved academic level	Ordered categorical (4 categories)	No missing data	3 indicator variables
Sex	Binary	No missing data	Binary
Economic status of the household	Ordered categorical (3 categories)	No missing data	2 indicator variables
Family structure	Binary	No missing data	Binary
Nationality	Binary	No missing data	Binary
Daily smoking	Binary	No missing data	Binary
Experience of severe problems	Binary	No missing data	Binary
Year of high school admission	Ordered categorical (9 categories)	No missing data	8 indicator variables
